# CRISPR knockout screens reveal JUN as the master mediator of resistance to MAPK inhibition in KRAS-mutant pancreatic cancer

**DOI:** 10.1186/s13046-025-03616-z

**Published:** 2026-01-22

**Authors:** Antonio Mulero-Sánchez, Astrid Bosma, Bonifiya Visuvasam, Niki Pouliopoulou, Marieke van de Ven, Natalie Proost, Manon Boeije, Cor Lieftink, Roderick Beijersbergen, Rene Bernards, Sara Mainardi

**Affiliations:** 1 Division of Molecular Carcinogenesis, Oncode Institute, The Netherlands Cancer Institute, Plesmanlaan 121, Amsterdam, 1066CX the Netherlands; 2 SOLTI Breast Cancer Research Group, Barcelona, Spain; 3 Mouse Clinic for Cancer and Aging Research, Preclinical Intervention Unit, The Netherlands Cancer Institute, Amsterdam, 1066CX the Netherlands; 4 Division of Molecular Carcinogenesis, The NKI Robotics and Screening Center, The Netherlands Cancer Institute, Plesmanlaan 121, Amsterdam, 1066CX the Netherlands

**Keywords:** Targeted therapy, Resistance, K-RAS, SHP2, ERK, JUN, Pancreatic cancer, RAS inhibitors

## Abstract

**Background:**

Pancreatic ductal adenocarcinoma (PDAC) is often driven by KRAS mutations, but inhibitors targeting the most frequent KRAS substitutions in PDAC are not yet approved in the clinic. We previously discovered that KRAS-mutant PDAC is sensitive to the combination of SHP2 and ERK inhibitors, recently investigated in the Phase I/Ib clinical trial NCT04916236. Lately, RAS(ON) multi-selective inhibitors have entered clinical development, representing a promise for mono or combination therapies in PDAC. However, resistance may arise even for combination therapies. Here, we aimed at anticipating mechanisms of resistance to SHP2 plus ERK or RAS(ON) multi-selective inhibitors.

**Methods:**

We performed a genome-wide CRISPR-KO screening, followed by four follow-up focused screenings, leading to the identification of resistance mediators, which were further validated through functional genetic and pharmacological experiments, both *in vitro* and *in vivo*.

**Results:**

Through unbiased CRISPR-based screenings, we identified mTOR and JUN hyperactivation as interconnected mechanisms that overcome MAPK suppression. Further investigation pointed at JUN as the most downstream resistance mediator, and indirect therapeutic target, using MAP2K4 inhibitors.

**Conclusions:**

Alterations in the PI3K/AKT/mTOR and JUN pathways can induce resistance to multiple combinations of MAPK pathway inhibitors, and may serve as biomarkers for sensitivity/resistance in clinical trials exploring such combinations in KRAS-mutant PDAC.

**Supplementary Information:**

The online version contains supplementary material available at 10.1186/s13046-025-03616-z.

## Background

Pancreatic cancer remains one of the deadliest malignancies worldwide, with a dismal 10% 5-year survival rate, largely due to late diagnosis and the limited efficacy of existing treatments [1], still widely relying on classic cytotoxic chemotherapy [2]. Targeted therapies, which have revolutionized treatment in other cancer types, have only marginally benefited pancreatic cancer, with poly ADP-ribose polymerase (PARP) inhibitors showing efficacy in a small subset of patients with homologous recombination deficiency[3]. However, these represent just 5–9% of pancreatic cancer cases, leaving the vast majority of patients without effective, genomically informed treatment options.

Mutations in the KRAS (Kirsten rat sarcoma) oncogene are present in more than 85% of pancreatic cancers, making it the most prevalent and critical oncogenic driver in this malignancy [4]. Therefore, KRAS has long been considered a prime therapeutic target, although due to its structure and biochemical properties, it was historically labeled as “undruggable.” Recently, this paradigm has shifted, with significant progress in the development of small molecule inhibitors that directly target KRAS [5]. Several KRAS-G12C-specific inhibitors—such as sotorasib and adagrasib—have gained FDA approval for KRAS-G12C-mutant non-small cell lung cancer (NSCLC), marking a major milestone [6–9]. However, KRAS-G12C mutations are rare in pancreatic cancer, where the most common mutation is KRAS-G12D, for which targeted inhibitors are still in early-phase clinical development [8, 10–12]. Pan-RAS or mutation-agnostic inhibitors, as well as RAS degraders, are also being explored as broader strategies, but none have yet received clinical approval [5, 13–16].

Given the challenges associated with direct KRAS inhibition, alternative strategies have focused on targeting critical nodes within the downstream MAPK (mitogen-activated protein kinase) signaling cascade, such as RAF (rapidly accelerated fibrosarcoma), MEK (mitogen-activated protein kinase kinase), and ERK (extracellular signal-regulated kinase) [17]. However, single-agent inhibition has generally yielded modest and short-lived responses, due to the complex network of feedback and compensatory pathways that can rapidly bypass a single-node blockade [18–20]. As a result, combination therapies targeting multiple components of the signaling network have emerged as a more effective approach [21]. Combinations of KRAS inhibitors with other agents such as SHP2 (Src homology region 2 (SH2)-containing protein tyrosine phosphatase-2), SOS1 (son of sevenless homolog 1), or EGFR (epidermal growth factor receptor) inhibitors have shown promise in preclinical models and early clinical trials [22, 23]. For instance, in KRAS-G12C-mutant colorectal cancer, the FDA has approved the use of KRAS-G12C inhibitors in combination with EGFR-targeting antibodies (adagrasib plus cetuximab or sotorasib plus panitumumab), illustrating the clinical potential of rationally designed combinations [24, 25]. Nevertheless, even such dual-targeted strategies are not immune to the problem of resistance.

Our group has recently contributed to this field by demonstrating, in preclinical models of pancreatic ductal adenocarcinoma (PDAC), that the combination of SHP2 inhibition (using RMC-4550) and ERK inhibition (using LY3214996) can achieve enhanced antitumor efficacy [26]. This strategy has recently been evaluated in the SHERPA Phase 1a/1b clinical trial (NCT04916236), which tested the clinical-grade SHP2 inhibitor RMC-4630 in combination with LY3214996 in patients with KRAS-mutant metastatic cancer. Despite these advances, early evidence and previous experience in other cancers suggest that resistance will likely emerge, even with dual inhibition.

Understanding how resistance develops in response to combined pathway inhibition is essential for optimizing therapeutic strategies. Identification of the molecular mechanisms underlying resistance can inform the development of predictive biomarkers, guide patient selection, and suggest new combinatorial approaches to overcome or delay resistance. In this study, we investigated potential mechanisms of resistance to combined SHP2 and ERK inhibition in KRAS-mutant pancreatic cancer. Additionally, we examined those resistance pathways in the context of an alternative dual therapy involving SHP2 and RAS(ON)-multi selective inhibitors [27]. By elucidating these resistance mechanisms, we hope to contribute to the discovery of biomarkers of resistance, and to the design of more effective, adaptive therapeutic strategies for patients with KRAS-driven pancreatic cancer.

## Methods

### Cell lines

Human PDAC cell lines YAPC-1 (KRASp.G12V; p53p.H179R; SMAD4p.R515fs*22), ASPC-1 (KRASp.G12D; p53p.C135fs*35; SMAD4p.R100T; CDKN2Ap.L78fs*41), Panc 10.05 (*KRASp.G12D; p53p.I255N*), Panc-1 (*KRASp.G12D; p53p.R273H*) and MiaPaCa-2 (*KRASp.G12C; p53p.R248W*; homozygous for *CDKN2A* deletion) were purchased from the American Type Culture Collection (ATCC). Mutational status of the cell lines was compiled from the ATCC, Catalogue of Somatic Mutations in Cancer (COSMIC; Welcome Trust Sanger Institute) and Cancer Cell Line Encyclopedia (CCLE, Broad Institute) databases. MiaPaCa-2 Resistant and Resistant_1 cells were generated by continuous exposure to RMC-4550 plus LY3214996 (both up to 2 μM) or RMC-4550 plus RMC-6236 (up to 5 nM) respectively. All PDAC cell lines were cultured in RPMI-1640 (Life Technologies), supplemented with 10% fetal bovine serum (FBS), penicillin/streptomycin (100 U/ml, 100 µg/ml, Life Technologies), and 2 mM L-glutamine (Thermo Fisher Scientific). For lentivirus production, HEK293T cells were cultured with Dulbecco's Modified Eagle Medium (DMEM) (GIBCO, 41966029) supplemented with 10% FBS, 5% penicillin/streptomycin and 2 mM L-glutamine. All cells were cultured at 37 °C in a humidified incubator with 5% CO2. The PDAC cell lines were authenticated by applying short tandem-repeat (STR) DNA profiling. Mycoplasma contamination was excluded via a polymerase chain reaction (PCR) -based method.

### Compounds and antibodies

LY3214996 was provided by Eli Lilly. RMC-4550 was provided by Revolution Medicines. RMC-6236 and SP-600125 were purchased from MedKoo®. HRX-0233 was provided by HepaRegeniX. AZD8055 (S1555) was purchased from Selleck Chemicals®. Antibodies against GAPDH (glyceraldehyde-3-phosphate dehydrogenase, #5174), S6RP (S6 rybosomal protein, #2217), p-S6RP (#2211 and #5361), RSK1 (ribosomal S6 kinase 1, #8408), p-RSK1 (#9344 and #8753), AKT (protein kinase B, #2920), PTEN (phosphatase and TENsin homolog deleted on chromosome 10, #9552), p-AKT (#4060), JUN (c-Jun, #2315), p-JUN (#3270) and PPP2R4 (protein phosphatase 2 A, regulatory subunit B, member 4, #3330) were purchased from Cell Signaling Technology. Antibodies against alpha-Tubulin (T9026) and vinculin (V9131) were purchased from Sigma-Aldrich.

### RNA sequencing

Cells were plated in 10 cm dishes at a density of 1,000,000 to 2,000,000 cells per plate, depending on growth rate. Cells were treated with the drugs of interest for 24 and 72 h. The library was prepared using the TruSeq RNA Sample Preparation kit (Illumina). On the RNASeq data for each timepoint a multifactor analysis with DESeq2 [28] was performed for PTEN mutant vs wildtype and treated vs untreated. From the results from this analysis, the DESeq2 test statistic for the interaction effect was used to sort the genes from positive to negative, where positive statistic values correspond to positive log2 fold change values and negative statistic values to negative log2 fold change values.

### Genome wide CRISPR screen

CRISPR (clustered regularly interspaced short palindromic repeats) screen was performed in triplicate with a coverage of 100x. For this, 260 millions Panc 10.05 cells were seeded. When attached, cells were infected with lentiviral particles containing the genome wide CRISPR knockout (KO) Brunello library [29] at an MOI (multiplicity of infection) of 0.3. Next, cells were selected with puromycin until control plates were empty. At this time, t = 0 reference control was collected and frozen. Remaining cells were divided in untreated or treated arms with either 2 or 4 µM of both RMC-4550 and LY3214996. After 8 doubling times untreated population was collected and frozen. Treated cells were collected after 23 days (low dose arm) or 58 days (high dose arm), to allow for sufficient expansion of the resistant colonies. Single guide RNA (sgRNA) sequences were then recovered by PCR and sequenced to determine their abundance. For sequence depth normalization, a relative total size factor was calculated for each sample by dividing the total counts of each sample by the geometric mean of all totals. All values within a sample were then divided by the respective relative total size factor and rounded off to integer values. A differential analysis between “treated” versus “untreated” condition was performed per sgRNA using DESeq2 [28]. The results of this analysis was used as input for an analysis on the gene level for enrichment, using MAGeCK’s robustrank algorithm (RRA) [30] which gives a test statistic, *P*-value and FDR (false discovery rate) value for enrichment of the sgRNAs of gene towards the top. In addition, we calculated a median log2 fold change per gene over the sgRNAs based on the DESeq2 output. Hits were selected based on FDR smaller or equal to 0.1 and median log2 fold change. All hits had log2 fold change greater or equal than 4.

### Generation of custom sgRNA library

For the design of the custom sgRNA library, targeting sgRNAs were designed using the Broad GPP sgRNA design portal. For the 167 genes selected from the genome-wide screen we designed 8 sgRNAs per gene. The controls, consisting of sgRNAs targeting essential genes (43 genes with 4 sgRNAs/gene), non-essential genes (47 genes with 4 sgRNAs/gene) or safe-targeting regions (233 sgRNAs) were designed as described previously [31]. The sgRNA sequences were ordered as a pool of oligonucleotides (Twist Biosciences) with flanking sequences to enable PCR amplification and Gibson assembly into pLentiGuide-Puro (pLG, addgene #52,963). The pooled oligo library was amplified using pLG_U6_foward 5'-GGCTTTATATATCTTGTGGAAAGGACGAAACACCG-3' and pLG-TRACR_Reverse 5'-GACTAGCCTTATTTTAACTTGCTATTTCTAGCTCTAAAAC-3'. The fragments were purified and cloned into pLG using Gibson assembly, as described by Morgens [32]. The representation and distribution of the custom sgRNA library was validated by next generation sequencing.

### Focused CRISPR resistance screen

When attached, cells were infected with lentiviral particles containing our customized library at an MOI (multiplicity of infection) of 0.3 and a coverage of 800x. Next, cells were selected with puromycin until control plates were empty. At this time, t = 0 reference control was collected and frozen. Remaining cells were divided in untreated or treated arms with 4 µM of both RMC-4550 and LY3214996. sgRNA sequences were recovered and sequenced as previously described for the genome-wide resistance screen.

### Interactomic analyses

Analyses for physical and functional interaction was done using the STRING software v11.5 providing manually the input list of genes [33].

### CRISPR–Cas9-mediated gene knockout

Cas9 (CRISPR-associated protein 9) expressing cell lines were obtained by lentiviral infection and selected either by fluorescence-associated cell sorting (FACS) of high Cas9-GFP (green fluorescence protein) levels (when the construct Addgene #63,592 was used) or with Blasticidine (when using the construct Addgene #73,310). Next, Cas9 levels were checked by western blot. CRISPR–Cas9-based *knock out (KO)* cell populations were obtained by lentiviral infection and selection with puromycin of sgRNAs targeting the different genes of interest. The following sgRNAs were used: PTEN-7 AGAGCGTGCAGATAATGACA; PTEN-8 (also named PTEN-2) AGCTGGCAGACCACAAACTG; PPP2R4-1 CTCGGAGACTCTGTACTCGA; PPP2R4-2 TGTGCCGTAGTCAATGCGCG. After selection, single-cell clones were picked and grown. Western blot analysis was used to check the protein expression levels of the KO cells.

### JUN overexpression

For the lentiviral transduction of the overexpression construct pLX304-Blast-V5-cJUN, 350 × 10^3^ cells per well were seeded in 6-well plates and incubated at 37 °C and 5% CO2 for 24 h to allow attachment. Thereafter, cells were incubated with virus in the presence of 1:1000 polybrene for at least 24 h, after which cells were selected using blasticydin (5 µg/ml), and finally used for further experiments.

### Long-term cell proliferation assays (colony formation)

Cells were cultured and seeded into 6-well plates at a density of 5–40 × 10^4^ cells per well, depending on growth rate, and were cultured in medium containing the indicated drugs for at least 2 weeks (medium was changed twice a week), unless differently stated. Alternatively, to explore a wider range of drug concentrations, 500 cells per well were cultured in 96 well plates for 10 days. After this, cells were fixed with 4% formaldehyde in PBS and stained with 0.1% crystal violet in water.

### Incucyte cell-proliferation assay and apoptosis assay

Indicated cell lines were seeded into 96-well plates at a density of 1,000–2,000 cells per well, depending on growth rate and the design of the experiment. Approximately 24 h later, drugs were added at the indicated concentrations using the HP D300 Digital Dispenser (HP). Cells were imaged every 4 h using the Incucyte ZOOM (Essen Bioscience). Phase-contrast images were analyzed to detect cell proliferation on the basis of cell confluence. For cell apoptosis, caspase-3/caspase-7 green apoptosis-assay reagent (Essen Bioscience) was added to the culture medium (1:1000), and cell apoptosis was analyzed on the basis of green-fluorescent staining of apoptotic cells.

### Protein lysate preparation and immunoblotting

To prepare analysis of cell lysates, cells were plated in complete medium. After attachment, cells were refreshed with medium containing the drugs of interest. At the desired time points, the cells were washed with cold-PBS and lysed in RIPA (radioimmunoprecipitation assay) buffer supplemented with Halt™ Protease & Phosphatase single-use inhibitors cocktail (100X) (78442) and HaltTM Protease single-use inhibitors cocktail (100X) (78430). Protein quantification was performed with the BCA (bicinchoninic acid) Protein Assay Kit (Pierce). The lysates were then resolved by electrophoresis in Bolt 4–12%Bis–Tris Plus Gels (Thermo Fisher Scientific) followed by standard western blotting.

### Xenografts

For the Panc10.05 PTEN KO xenograft experiment, 5 × 10^6^ cells (clone 2.1.4) were subcutaneously injected into the right flank of Nod-Scid-gamma (NSG) mice. When tumors reached 200–250 mm^3^, mice were randomly assigned into cohorts (n = 8 mice per group) and treated daily by oral gavage with either vehicle, HRX-b (250 mg/kg), RMC4550 (10 mg/kg) + LY3214996 (100 mg/kg), or the triple combination of RMC4550 (10 mg/kg) + LY3214996 (100 mg/kg) + HRX-b (250 mg/kg). The tumor volume was monitored over time and never allowed to reach > 1500 mm^3^.

### Immunohistochemistry

For immunohistochemical analysis, formalin-fixed paraffin-embedded tumor samples from the previously reported xenograft experiment [26] were probed with p-S6RP S235/236 antibody (Cell Signaling #2211 at 1:400).

### Statistics

All *in vitro* data are expressed as averages from at least three technical replicates ± standard deviation (SD) or standard error of the mean (SEM) as indicated, and they have been independently reproduced at least twice with similar results.

## Results

### A two-step CRISPR knockout screen identifies mediators of resistance to the combination of SHP2 and ERK inhibitors in PDAC

**Fig. 1:**
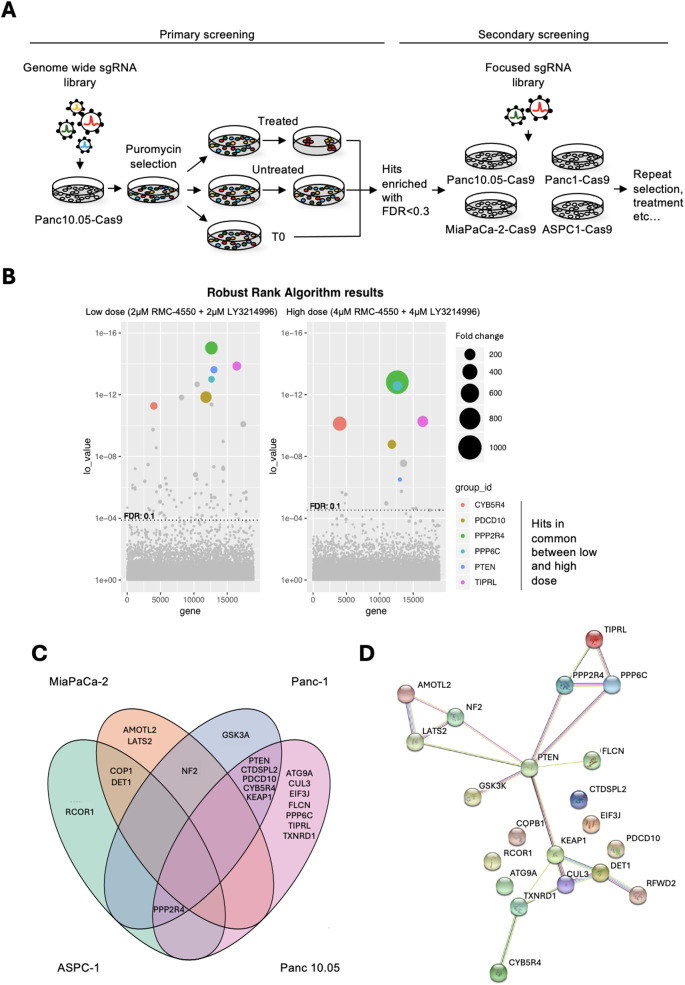
CRISPR genetic screens uncover mediators of resistance to SHP2 plus ERK inhibition in KRAS-mutant PDAC. **A**: schematic representation of the workflow, from primary to secondary resistance screenings. **B**: bubble plots depicting the results of the primary genome-wide resistance screen in Panc 10.05. The colored bubbles represent genes significantly enriched (FDR < = 0.1) in both low-dose (left panel) and high-dose (right panel) treated arms. The size of the bubbles correlates with fold change enrichment. **C**: Venn diagram highlighting the genes significantly enriched in the focused screenings, and how they distribute and overlap between the different cell lines. MiaPaCa-2 oval is orange, ASPC-1 is green, Panc-1 is blue and Panc 10.05 is pink. **D**: STRING interactomic analysis for all the hits obtained in the four focused screenings

To systematically identify mediators of resistance to the combined inhibition of SHP2 and ERK in PDAC, we performed a genome-wide CRISPR knockout (KO) screen in the Panc 10.05 cells (Fig. 1A), using either a “low-dose” (2 μM of the SHP2 inhibitor RMC-4550 plus 2 μM of the ERK inhibitor LY3214996) or a “high-dose” (4 μM of each) of the drugs. Both concentrations were effective in inhibiting the proliferation of Panc 10.05, as well as other PDAC cell lines (ASPC-1, MiaPaCa-2, Panc-1, and YAPC-1), in Incucyte assays (Supplementary Fig. 1 A, Additional files 1 and 2). After 23 days (for the “low-dose”) or 58 days (for the “high-dose”) of treatment, drug-resistant colonies had proliferated enough to allow genomic DNA extraction and sequencing. Genes were considered hits if multiple sgRNAs targeting them were enriched at least 16-fold, with a false discovery rate (FDR) lower than 0.1. Overall, a lower number of hits was identified in the “high dose” arm, in line with the more stringent selective conditions (Fig. 1B). Consistent with the contribution of CRISPR-mediated perturbations to resistance, CRISPR library-transduced cells formed larger colonies than non-transduced controls (Supplementary Fig. 1B, Additional files 1 and 2). To note, only six hits were shared between the “low- “ and “high-dose” screens: PTEN (phosphatase and TENsin homolog deleted on chromosome 10), PPP6C (protein phosphatase 6 catalytic subunit), PPP2R4 (protein phosphatase 2 A regulatory subunit 4), TIPRL (TOR signaling pathway regulator-like), CYB5R4 (cytochrome b5 reductase 4), and PDCD10 (programmed cell death 10) (Fig. 1B).

To refine our findings, we built a focused secondary sgRNA library targeting 167 genes that were enriched > 16-fold (FDR < 0.3) in the “low-dose” arm of the primary screen (Fig. 1A). This secondary library was used in a high-coverage (800 ×) screen in four KRAS-mutant PDAC cell lines (ASPC-1, MiaPaCa-2, Panc-1, and Panc 10.05) under “high-dose” conditions (4 μM RMC-4550 plus 4 μM LY3214996). A smaller number of hits could be identified in MiaPaCa-2 and ASPC-1 cells compared to Panc 10.05 and Panc-1 (Fig. 1C), suggesting that those cell lines may be substantially different from the Panc 10.05 that was used in the primary screen. Interestingly, DET1 (de-etiolated homolog 1) and COP1 (constitutive photomorphogenesis protein 1), members of the CUL4 (cullin 4) -DDB1 (DNA damage binding protein 1) ubiquitin ligase complex known to regulate JUN and MAPK signaling [34], emerged as shared resistance genes in both MiaPaCa-2 and ASPC-1. These genes have also been implicated in resistance to MAPK pathway inhibitors in melanoma and gastrointestinal stromal tumors [35]. Several resistance hits overlapped across Panc 10.05 and Panc-1, including PTEN, CYB5R4, and PDCD10. To note, PPP2R4 was enriched in three of four cell lines (absent only in MiaPaCa-2), but individual sgRNA validation failed to confirm its role in resistance (Supplementary Fig. 1 C, Additional files 1 and 2). We performed STRING-based interactome analysis of all the hits identified through the secondary screening (therefore originally identified in Panc10.05 and subsequently in at least another cell line). This revealed PTEN as a central hub connecting several resistance-associated nodes (Fig. 1D), reinforcing its functional importance. Therefore, we prioritized PTEN for further validation and mechanistic investigation.

### PTEN knockout drives resistance to the combination of SHP2 and ERK inhibitors in PDAC

**Fig. 2:**
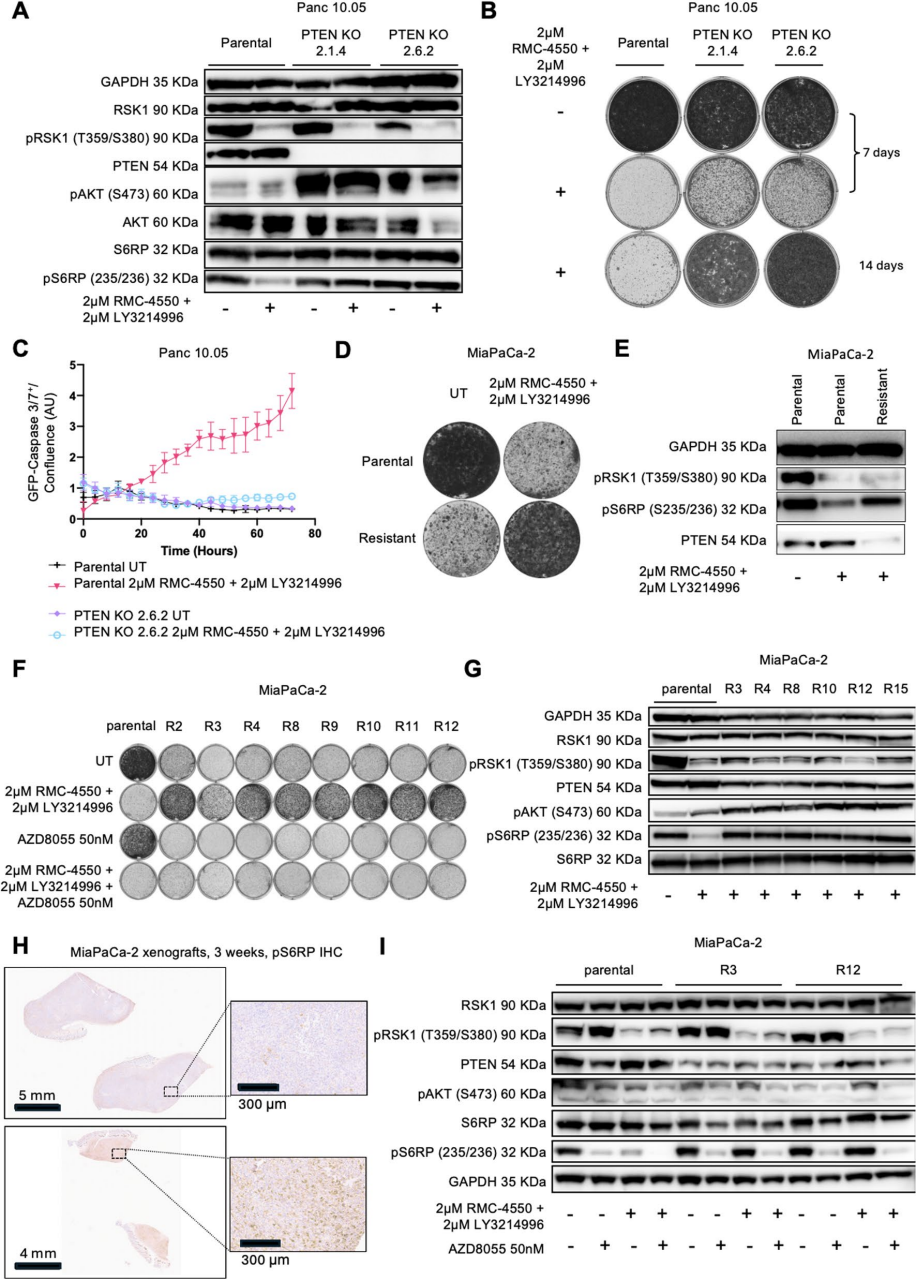
Resistance to SHP2i + ERKi is induced by PTEN knockout or spontaneous activation of the AKT/mTOR pathway. **A**: western blot of Panc 10.05 parental and PTEN KO clones 2.1.4 and 2.6.2, untreated or treated with 2 μM LY3214996 + 2 μM RMC-4550 for 48 h. **B**: long term proliferation assay of Panc 10.05 parental or PTEN KO clones, untreated or treated with 2 μM LY3214996 + 2 μM RMC-4550. After 7 or 14 days, cells were fixed and stained with crystal violet. **C**: GFP-Caspase 3/7 apoptosis assay depicting GFP levels over time in Panc 10.05 parental and PTEN KO clone 2.6.2. Cells were untreated (UT) or treated with 2 μM LY3214996 + 2 μM RMC-4550 and monitored through Incucyte. Plot represents the average of n = 3 replicates, error bars represent standard deviations. **D**: long term proliferation assay of MiaPaCa-2 parental (20 K cells seeded, 10 days cultured) and resistant heterogenic populations (40 K cells seeded, 18 days cultured) in the absence (UT) or presence of 2 μM LY3214996 + 2 μM RMC-4550. **E**: western blot of MiaPaCa-2 parental cells untreated and MiaPaCa-2 parental and resistant cells treated with 2 μM LY3214996 + 2 μM RMC-4550 for 48 h. **F**: long term proliferation assay of MiaPaCa-2 parental cells and 8 MiaPaCa-2 resistant (R) single-cell clones, untreated (UT), or treated with: 2 μM LY3214996 + 2 μM RMC-4550, 50 nM AZD8055 or the triple combination (2 μM LY3214996 + 2 μM RMC-4550 + 50 nM AZD80555) for 12 days. **G**: western blot of MiaPaCa-2 parental untreated and MiaPaCa-2 parental and 6 different MiaPaCa-2 resistant (R) monoclonal polulations treated for 48 h with 2 μM LY3214996 + 2 μM RMC-4550. **H**: pS6RP immunohistochemistry on tumor tissue from MiaPaCa-2 xenografts treated with vehicle or the combination of RMC-4550 (10 mg/kg) + LY3214996 (100 mg/kg) for 3 weeks. Images representative of 3 independently stained sections. **I**: western blot of MiaPaCa-2 parental and resistant single-cell clones R3 and R12 untreated or treated for 48 h with either AZD8055 50 nM, 2 μM LY3214996 + 2 μM RMC-4550 or the triple combination. In A, B, D, E, F, G, I, images are representative of at least 3 independent experiments giving similar results

To validate PTEN as a mediator of resistance, we generated PTEN knockout monoclonal lines using lentiviral transduction of sgRNAs in GFP-Cas9-expressing Panc 10.05 and Panc-1. Western blotting confirmed the absence of PTEN protein in Panc10.05 and Panc1 selected clones (Panc 10.05 clones 2.1.4 and 2.6.2; Panc-1 clones 7.8 and 8.6) (Fig. 2A, Supplementary Fig. 2A, Additional files 1 and 2). In long-term proliferation assays, the PTEN KO clones showed resistance to RMC-4550 + LY3214996, while parental cells remained sensitive (Fig. 2B, Supplementary Fig. 2B, Additional files 1 and 2). Similar results were obtained in ASPC-1 PTEN KO polyclonal populations (sgRNA 7 and sgRNA 8) (Supplementary Fig. 2C-D). Overall, loss of PTEN promotes resistance to SHP2 + ERK inhibition, likely via upregulation of the PI3K (phosphoinositide 3-kinase) -AKT-mTOR (mammalian target of rapamycin) survival pathway.

Because SHP2 + ERK inhibition induces apoptosis in PDAC cells, as we previously demonstrated [26], we asked whether PTEN loss suppresses drug-induced cell death. Using a Caspase-3/7 GFP reporter, we found that PTEN KO Panc 10.05 clone 2.6.2 failed to activate apoptosis following SHP2 + ERK inhibition (Fig. 2C), in contrast with the parental line, where caspase activation was robust. Similar findings were obtained in Panc-1 PTEN KO 7.8 and in Panc 10.05 PTEN KO 2.1.4 and 2.6.2 after 72 h of treatment (Supplementary Fig. 3A-B, Additional files 1 and 2). The GFP signal in clone 2.1.4 was elevated at baseline, likely due to higher Cas9-GFP expression (Supplementary Fig. 3 C, Additional files 1 and 2), but no apoptotic induction was observed in response to treatment. These findings suggest that PTEN loss blocks apoptosis downstream of MAPK inhibition.

We then evaluated signaling pathway activity by immunoblotting. PTEN KO led to increased phosphorylation of AKT (pAKT, Ser473) in Panc 10.05, Panc-1 and ASPC-1 (Fig. 2A, Supplementary Fig. 2A, C, Additional files 1 and 2), indicating PI3K-AKT-mTOR pathway activation. MAPK pathway inhibition remained intact upon treatment with SHP2 plus ERK inhibitors in all cells, as shown by reduced pRSK. However, phosphorylation of S6 ribosomal protein (pS6RP, Ser235/236), a downstream target of mammalian target of rapamycin complex 1 (mTORC1), was sustained in Panc 10.05 and ASPC-1 PTEN KO cells even upon SHP2 + ERK inhibition, whereas it was suppressed in treated parental cells (Fig. 2A, Supplementary Fig. 2C, Additional files 1 and 2). Notably, treatment of Panc-1 parental cells did not affect the phosphorylation of S6RP as much. However, PTEN knockout Panc-1 cells showed increased levels of Ser235/236 S6RP phosphorylation both in the treated and untreated conditions, suggesting that increased activity of mTOR is driving survival and proliferation also in this model (Supplementary Fig. 2 A, Additional files 1 and 2). This suggests that mTOR signaling persists in PTEN-deficient cells in the absence of MAPK reactivation, driving survival in the presence of SHP2 plus ERK inhibitors.

### Spontaneous resistance also involves activation of the PI3K-AKT-mTOR pathway

To determine whether similar mechanisms occur during spontaneous resistance, we exposed parental MiaPaCa-2, Panc 10.05, and ASPC-1 cells to chronic 2 μM RMC-4550 + 2 μM LY3214996. After prolonged exposure (> 2 months), resistant colonies emerged only in MiaPaCa-2. These resistant cells proliferated in the presence of the drugs, while parental cells remained sensitive (Fig. 2D). Notably, resistant cells showed decreased growth upon drug withdrawal, suggesting a drug-holiday-like effect [36]. Similar to what observed in PTEN KO cells, western blot analysis excluded a reactivation of the MAPK pathway in the spontaneously resistant MiaPaCa-2 cells, as indicated by low pRSK levels, comparable to the parental cells treated with SHP2 and ERK inhibitors (Fig. 2E). Importantly, PTEN protein levels were reduced, and pS6RP levels were elevated, indicating reactivation of the PI3K-AKT-mTOR pathway in the spontaneously resistant cells. Thus, spontaneous resistance recapitulates the signaling features of PTEN KO, even though PTEN loss in this case may be partial or regulated at the transcriptional or post-translational level.

To further dissect the heterogeneity of resistance, we derived monoclonal lines from the resistant MiaPaCa-2 polyclonal population. All eight clones retained resistance to SHP2 + ERK inhibition and exhibited drug addiction, as drug withdrawal suppressed their proliferation (Fig. 2F). Immunoblots confirmed that the MAPK pathway remained inhibited (low pRSK), while pAKT and pS6RP levels were elevated in all resistant clones (Fig. 2G). PTEN protein was only moderately reduced, suggesting that partial suppression of PTEN or parallel mechanisms drive PI3K pathway activation. Overall, only one main mechanism of resistance to SHP2 + ERK inhibition could be identified, which discards the reactivation of the MAPK pathway and implies triggering activation of the parallel PI3K-AKT-mTOR axis.

We next asked whether this mechanism arises *in vivo*. MiaPaCa-2 xenograft tumors treated with RMC-4550 (10 mg/kg) and LY3214996 (100 mg/kg) regressed initially but ultimately failed to fully resolve [26]. Immunohistochemistry showed increased pS6RP staining in 3 weeks-treated tumors compared to vehicle controls (Fig. 2H, Supplementary Fig. 3D, Additional files 1 and 2), suggesting mTOR activation during acquired resistance *in vivo*.

Since all resistant models showed mTOR activation, we hypothesized that mTOR inhibition might resensitize resistant cells. Treatment of MiaPaCa-2 resistant monoclonal lines with the mTOR inhibitor AZD8055 (50 nM) suppressed proliferation, while parental MiaPaCa-2 cells were unaffected (Fig. 2F). A triple combination of SHP2, ERK, and mTOR inhibitors suppressed growth in both parental and resistant cells. Immunoblotting showed decreased pRSK and pS6RP in MiaPaCa-2 parental cells upon 48 h treatment with the dual or triple therapy (Fig. 2I), as previously observed in Fig. 2G. On the contrary, the MiaPaCa-2 resistant single-cell clones exhibited increased pS6RP levels, upon combined SHP2 + ERK inhibition, which was suppressed by either the triple combination, or mTOR inhibitor monotherapy. Interestingly, pRSK levels were higher in MiaPaCa-2 resistant monoclonal lines upon drug withdrawal (untreated condition) as compared to the untreated parental cells. Yet, long term proliferation assays showed growth impairement by drug withdrawal (Fig. 2F), suggesting that concomitant hyperactivation of both mTOR and MAPK pathways might be detrimental for KRAS mutant PDAC cells.

### Suppressing the mTOR pathway only partially prevents resistance to LY3214996 + RMC-4550

To test if mTOR inhibition could prevent resistance to the inhibition of SHP2 and ERK, we performed long-term proliferation assays in the presence of RMC-4550 + LY3214996 (2 μM each) ± AZD8055 (25 or 50 nM). While in YAPC-1 and Panc-1 resistance to SHP2 + ERK inhibitors was already present at the short timepoint (10–14 days, until confluency of the untreated control), resistant colonies of MiaPaCa-2, Panc10.05 and ASPC-1 cells were only evident after 50 days (Supplementary Fig. 4, Additional files 1 and 2). Importantly, the addition of low doses of AZD8055 was able to impair resistance in ASPC-1, Panc 10.05 and YAPC-1, but not in MiaPaCa-2 and Panc-1, implying that mTOR-independent mechanisms may contribute to resistance in some models.

### The mTOR pathway drives JUN activation and resistance

**Fig. 3:**
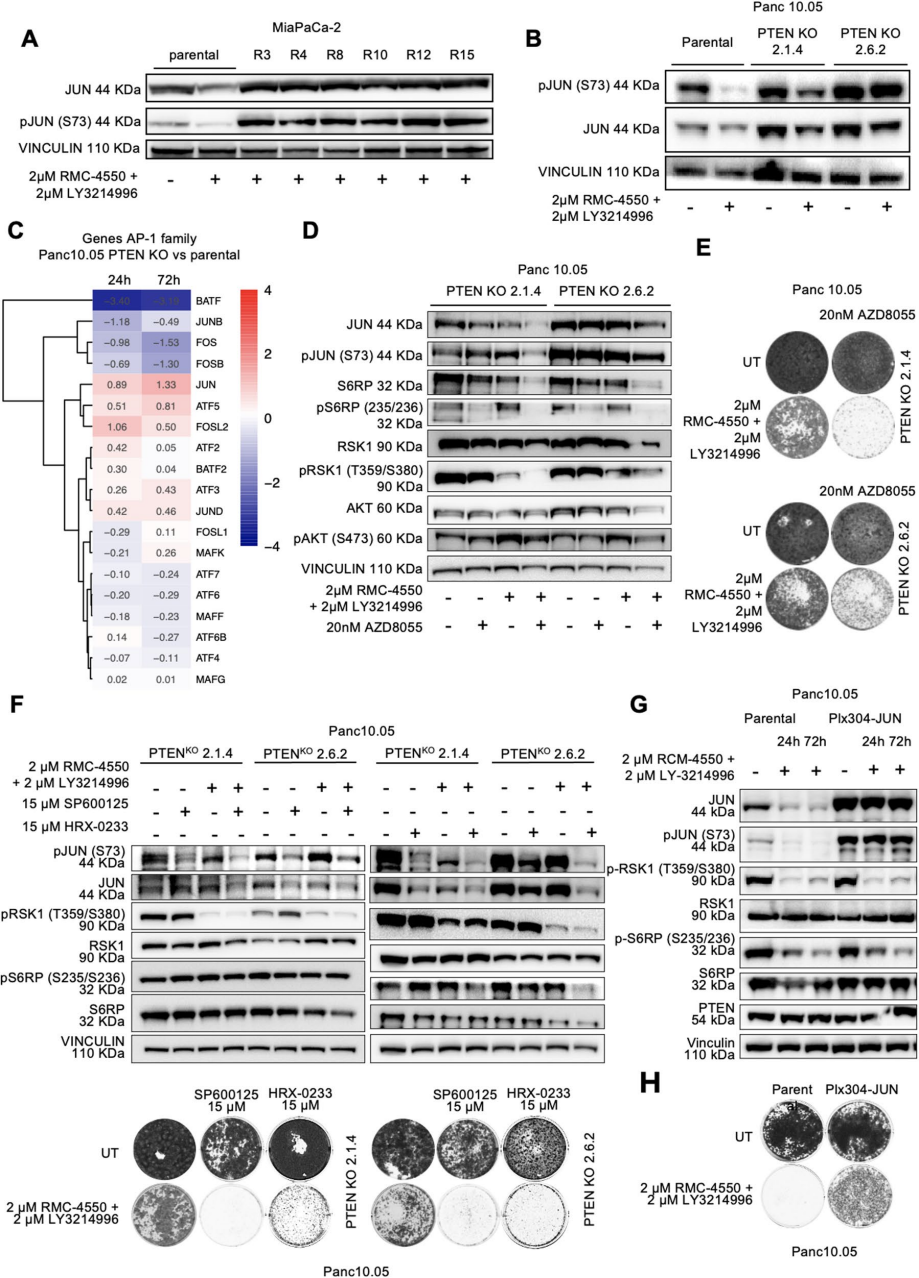
JUN is the most downstream mediator of resistance to SHP2 + ERK inhibition in KRAS-mutant PDAC. **A**: western blot of MiaPaCa-2 parental untreated and MiaPaCa-2 parental and 6 different MiaPaCa-2 resistant (R) single-cell clones treated for 48 h with 2 μM LY3214996 + 2 μM RMC-4550. **B**: western blot of Panc 10.05 parental and PTEN KO clones 2.1.4 and 2.6.2, untreated or treated with 2 μM LY3214996 + 2 μM RMC-4550 for 48 h. **C**: heatmap of gene expression of AP-1 family members in Panc 10.05 PTEN KO 2.1.4 and 2.6.2 clones relative to the parental Panc 10.05 (Log2FC). Cells were collected after 24 h or 72 h. **D**: western blot of Panc 10.05 PTEN KO clones 2.1.4 and 2.6.2 untreated or treated with 20 nM of the mTOR inhibitor AZD8055, 2 μM LY3214996 + 2 μM RMC-4550, or the triple combination for 72 h. **E**: long term proliferation assay of Panc 10.05 PTEN KO clones 2.1.4 (upper panel) and 2.6.2 (lower panel). Cells were left untreated or treated for 14 days with 20 nM AZD8055, 2 μM LY3214996 + 2 μM RMC-4550, or the triple combination. **F**: western blot (upper panel) and long term proliferation assay (lower panel) of Panc10.05 PTEN KO clones 2.1.4 and 2.6.2 untreated, treated with the JNK inhibitor SP600125 (15 μM), the MAP2K4 inhibitor HRX-0233 (15 μM), 2 μM LY3214996 + 2 μM RMC-4550, or one of the two triple combinations (LY3214996 + RMC-4550 + SP600125 or LY3214996 + RMC-4550 + HRX-0233) for 72 h or 14 days. **G**: western blot of Panc 10.05 parental and JUN overexpressing (Plx304-JUN) cells untreated or treated with 2 μM LY3214996 + 2 μM RMC-4550 for 24 h or 72 h. **H**: long term proliferation assay of Panc 10.05 parental and JUN overexpressing cells, untreated or treated with 2 μM LY3214996 + 2 μM RMC-4550 for 14 days. In panels A, B, D, E, F, G, H, images are representative of at least 3 independent experiments giving similar results

In our focused CRISPR screen, we also identified DET1 and COP1 (alias RFWD2) as resistance hits in MiaPaCa-2 and ASPC-1 (Fig. 1C). The two proteins form a E3 ubiquitin ligase complex that regulates JUN stability, and are known to mediate sensitivity to MEK inhibitors in KRAS mutant colorectal cancer [34]. We thus investigated whether JUN signaling is involved in resistance to SHP2 + ERK inhibitors in PDAC. Western blotting showed increased total and pJUN in MiaPaCa-2 resistant clones compared to treated parental cells (Fig. 3A). Together with pS6RP and pAKT upregulation (Fig. 2G), this indicates a possible cooperation or convergence of the PI3K-AKT-mTOR pathway and the JNK (c-Jun N-terminal kinase) -JUN pathway in driving resistance to the combined SHP2 and ERK inhibition. Supporting this notion, PTEN KO Panc 10.05 and ASPC-1 cells exhibited elevated pJUN levels (Fig. 3B and Supplementary Fig. 2 C, Additional files 1 and 2), which were not suppressed by SHP2 + ERK inhibition, in contrast with the parental counterpart where pJUN levels decrease in response to the treatment. Furthermore, RNA-seq analysis in Panc 10–05 PTEN KO cells revealed a broader dysregulation of the AP-1 (activator protein 1) transcription factor network. In PTEN KO cells, JUN was significantly upregulated, and the repressive AP-1 family member BATF (basic leucine zipper ATF-like transcription factor) [37] was downregulated (Fig. 3C), indicating a pro-proliferative shift in AP-1 activity. Notably, FOS (AP-1 transcription factor subunit) remained low, suggesting FOS-independent JUN activation.

To further investigate whether mTOR activity following PTEN loss is necessary for JUN upregulation and resistance, we treated Panc 10.05 PTEN KO clones with the mTOR inhibitor AZD8055, either alone or in combination with SHP2 and ERK inhibitors. The triple combination induced, after 72 h, a profound downregulation not only of the mTOR target S6RP, but also of total and phosphorylated JUN (Fig. 3D), while restoring sensitivity to SHP2 + ERK inhibitors in long term colony formation assays (Fig. 3E).

To additionally evaluate whether JUN upregulation following PTEN deletion and subsequent mTOR hyperactivation, is functional to the resistance phenotype of PTEN KO clones, we performed indirect pharmacological inhibition of JUN by targeting two nodes upstream in the pathway: the JUN kinase JNK (with SP600125) and the JNK kinase mitogen-activated protein kinase kinase 4 MAP2K4 (with HRX-0233). Both agents reduced pJUN (Fig. 3F, upper panel) and restored sensitivity to SHP2 + ERK inhibitors in PTEN KO cells (Fig. 3F lower panel), despite persistent S6RP phosphorylation, suggesting that JUN is the most downstream mediator of resistance to MAPK inhibition in PDAC models. Similar results were obtained using a Panc-1 PTEN KO clone (Supplementary Fig. 5 A, Additional files 1 and 2).

By using a reverse approach, we next overexpressed JUN in Panc10.05 (Fig. 3G, H) and Panc-1 (Supplementary Fig. 5 B, Additional files 1 and 2): this increased pJUN and conferred resistance to SHP2 + ERK inhibition, without restoring MAPK signaling, as judged by low levels of pRSK and pS6RP. Overall, our data indicate that JUN activation downstream of mTOR is both necessary and sufficient for resistance to MAPK inhibition in KRAS-mutant PDAC.

### MAP2K4 inhibition restores sensitivity to the combination of SHP2 and ERK inhibitors in vivo

**Fig. 4:**
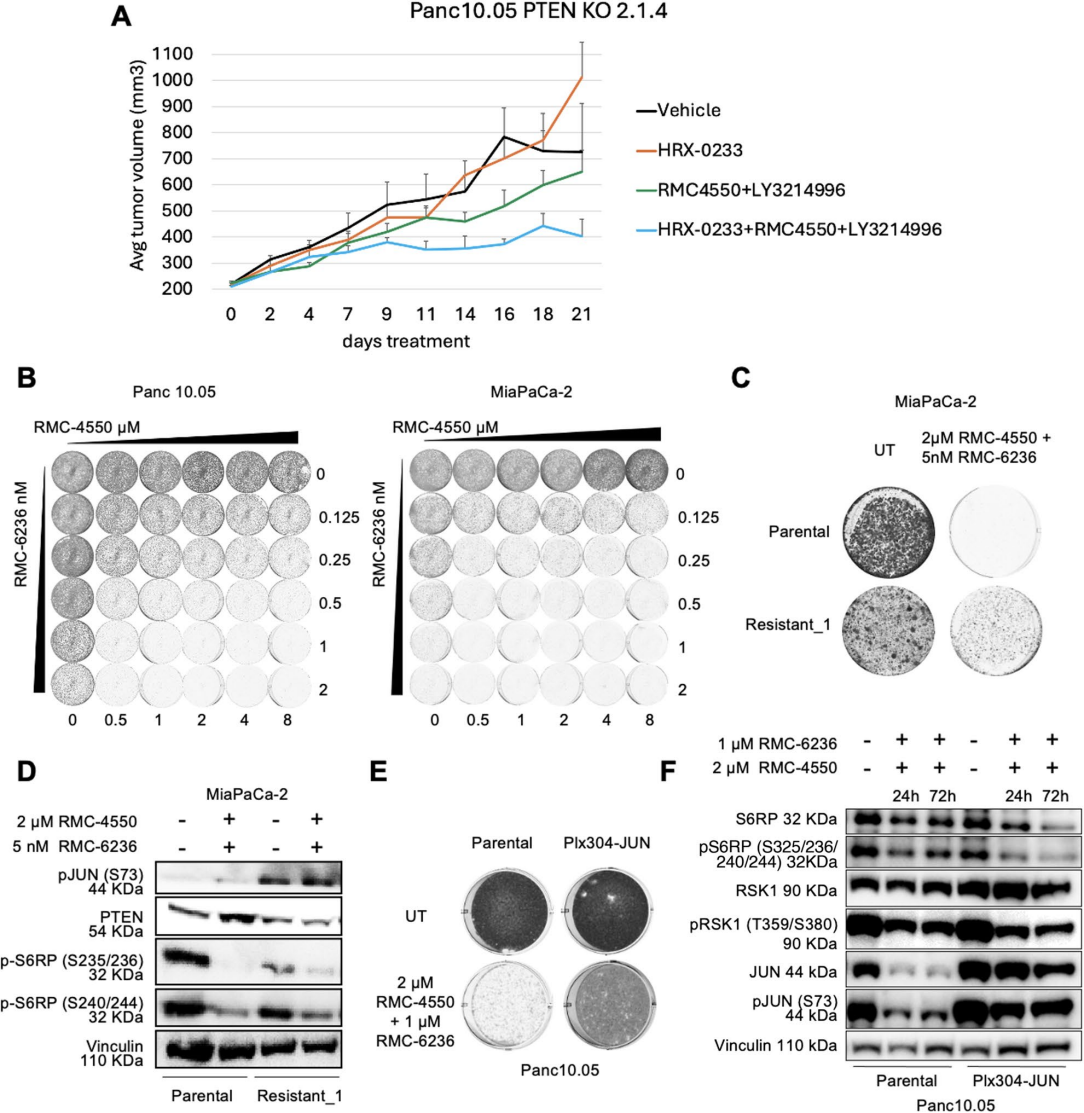
JUN as indirect *in vivo* target and mediator of resistance also to SHP2+RAS inhibitors. A: xenograft experiment for the *in vivo* evaluation of HRX-0233 as an antagonist of resistance to the combination of RMC-4550 +LY3214996. Resistant Panc 10.05 PTEN KO 2.1.4 (5 × 106) were subcutaneously injected into the right flank of NSG mice. When tumors reached 200–250 mm3, mice were randomly assigned into cohorts (n= 8 mice per group) and treated by oral gavage with inhibitors or vehicle according to treatment schedule for 21 days. Mean tumor volumes ± s.e.m. are shown. B: long term proliferation assay of Panc 10.05 and MiaPaCa-2 cells performed in a matrix of increasing concentrations of SHP2 inhibitor (RMC-4550) and RAS(ON) multi-selective inhibitor (RMC-6236) and their combination for 6 days. At the end, cells were fixed and stained with crystal violet. C: long term proliferation assay of MiaPaCa-2 parental and Resistant_1 (to SHP2 plus RAS inhibitors) heterogenic populations cultured either in the absence (UT) or presence of 2μM RMC-4550 +5nM RMC-6236. D: western blot of MiaPaCa-2 parental or Resistant_1 populations either untreated or treated with 2μM RMC-4550 +5nM RMC-6236. E: long term proliferation assay of Panc 10.05 parental and JUN overexpressing (Plx304-JUN) cells, untreated or treated with 2μM RMC-4550 +1μM RMC-6236 for 14 days. F: western blot of Panc 10.05 parental and JUN overexpressing (Plx304-JUN) cells either untreated or treated with the combination of 2μM RMC-4550 +1μM RMC-6236 for 24h or 72h hours. In panels B, C, D, E, F, images are representative of at least 3 independent experiments giving similar results

To assess whether indirectly targeting JUN could reverse resistance to MAPK inhibitors *in vivo*, we tried to obtain xenografts from the MiaPaCa-2 spontaneous resistant population. However, these cells failed to form tumors in NSG immunocompromised mice, suggesting loss of tumorigenic potential (Supplementary Fig. 6, Additional files 1 and 2). As an alternative, xenografts were established using Panc 10.05 PTEN KO cells, which maintained tumorigenicity. PTEN KO tumors displayed resistance to RMC-4550 + LY3214996, but the addition of the MAP2K4 inhibitor HRX-0233—which had no impact as monotherapy—restored growth control (Fig. 4A). These results support the notion that JUN inhibition may offer a viable therapeutic strategy to overcome MAPK inhibitor resistance in KRAS-mutant PDAC.

### JUN also mediates resistance to RAS(ON) inhibitors

Having established that JUN upregulation drives resistance to SHP2 + ERK inhibition downstream in the MAPK cascade, we next asked whether this mechanism would extend to more proximal blockade of MAPK signaling. Direct RAS inhibition has recently emerged as a promising strategy to overcome limitations of downstream kinase inhibitors in KRAS-mutant tumors, where ERK inhibitors can produce incomplete or transient pathway suppression [38]. Given this context, and the accelerated development of next-generation RAS(ON) multi-selective inhibitors, we reasoned that testing whether JUN also confers resistance to SHP2 + RAS(ON) inhibition would reveal whether JUN functions as a broader resistance node. Novel RAS(ON) multi-selective inhibitors, such as RMC-7977 and its clinical derivative RMC-6236, directly block both wild-type and mutant KRAS in the active GTP-bound state, offering a more upstream and potentially more durable block of MAPK signaling in PDAC models where KRAS G12C mutations are rare [39]. Motivated by these mechanistic advantages, we evaluated the effect of combining the SHP2 inhibitor RMC-4550 with RMC-6236 in MiaPaCa-2 and Panc 10.05 cell lines, and found robust synergy across a concentration range (Fig. 4B) comparable to the SHP2/ERK combination [26] (Supplementary Fig. 7 A, Additional files 1 and 2). To study resistance to this alternative combination, new MiaPaCa-2 spontaneous resistant cell lines (Resistant_1) were generated via prolonged exposure to 2 μM RMC-4550 + 5 nM RMC-6236. These Resistant_1 cells developed a less pronounced resistance phenotype than cells resistant to SHP2 + ERK inhibition (Fig. 4C vs. Figure 2D), suggesting that the SHP2 + RAS(ON) combo may delay resistance more effectively. Moreover, Resistant_1 lines retained proliferation after drug withdrawal, unlike previous resistant cells which displayed a drug-holiday-like effect.

Despite phenotypic differences, PTEN was also downregulated in Resistant_1 cells (Fig. 4D), yet this was not accompanied by mTOR pathway activation, as indicated by low pS6RP levels in response to the treatment. Yet, pJUN was markedly elevated, likely in a mTOR-independent fashion, suggesting a central role for JUN in mediating resistance not only to SHP2 + ERK inhibitors but also to SHP2 + RAS(ON) inhibitors.

To validate this, JUN-overexpressing Panc 10.05 and Panc-1 lines were tested in long-term colony formation and western blot assays. JUN overexpression was sufficient to drive cell proliferation in the presence of RMC-4550 + RMC-6236 without restoration of MAPK signaling, as indicated by low levels of pRSK and pS6RP (Fig. 4E–F; Supplementary Figs. 7B–C, Additional files 1 and 2).

Overall, our findings establish JUN as a convergent resistance node in KRAS-mutant PDAC, mediating resistance to both SHP2 + ERK and SHP2 + RAS(ON) combination therapies.

## Discussion

In this study, we used CRISPR functional genomic screenings to uncover mechanisms of resistance to the combination of SHP2 and ERK inhibition in KRAS-mutant PDAC, which is currently under early phases clinical investigation. Our screens collectively highlight both previously recognized and novel contributors to resistance to combined MAPK inhibition, emphasizing the adaptability of signaling networks under therapeutic pressure even in the presence of a double blockade, and unveiling potential vulnerabilities for further combination strategies.

Interestingly, while previously identified mechanisms of resistance to various MAPK inhibitors (including direct RAS inhibitors) as monotherapy, almost invariably implied a re-activation of the MAPK pathway [40–47], here we observe that MAPK reactivation does not occur in the presence of a two-nodes combined inhibition. Nevertheless, resistance to combined therapy can still emerge through alternative mechanisms leading to a state where mTOR signaling and JUN activity enable the cells to survive and grow under sustained MAPK pathway inhibition.

One of the most robust resistance mechanisms we identified involves loss or downregulation of the tumor suppressor PTEN. Our data show that PTEN deletion allows cells to maintain an active PI3K-AKT-mTOR survival signaling pathway, therefore avoiding apoptosis, even in the presence of the profound and sustained MAPK pathway suppression induced by the combined SHP2 and ERK inhibition. This observation resonates with previous reports demonstrating that PTEN-loss drives resistance to receptor tyrosine kinase (RTK) and MAPK pathway inhibitors (as monotherapies) in a range of tumors, by unlocking parallel signaling cascades [48–50]. Our results further highlight JUN upregulation as a key cooperator in resistance upon PTEN-loss. We show that PTEN KO not only maintains mTOR activity but also drives a pro-tumorigenic AP-1 transcriptional program, which includes elevated expression and activity of JUN. Importantly, this phenomenon could be reversed by pharmacological mTOR inhibition, which dampened both mTOR signaling and JUN expression, thereby resensitizing resistant cells. This highlights a previously unappreciated link between mTOR and JUN in resistance mechanisms and resonates with growing literature demonstrating the role of JUN/AP-1 factors in resistance to targeted therapies [51, 52].

While mTOR readouts are frequently upregulated upon resistance, adding mTOR inhibitors alongside SHP2 and ERK inhibition provided incomplete prevention of resistance in some models (MiaPaCa-2 and Panc-1). Moreover, our data indicate that JUN upregulation and resistance may not be fully mTOR-dependent, suggesting co-existing regulation mechanisms. Additional hits identified in our resistance screens, include DET1 and COP1, components of a Cullin 4 (CUL4)-RING E3 ligase complex previously shown to regulate JUN stability [53, 54]. This also resonates with previous reports demonstrating that DET1 and COP1 deletion stabilises JUN and drives resistance to RAF and MEK inhibitors in melanoma and gastrointestinal stromal tumors [35, 52]. Our data suggest this resistance route could be extended to KRAS-mutant PDAC under SHP2 and ERK inhibitor pressure. Importantly, this further highlights JUN as a convergence node, downstream of most resistance mechanisms.

We thus further explored whether JUN could represent an acquired vulnerability of the resistant cells. We found that indirect targeting of JUN, especially using small molecule inhibitors of the JNK activator MAP2K4 (HRX-0233), restores sensitivity to SHP2 + ERK inhibition *in vivo*, offering an actionable resistance-reversal strategy. This proof-of-concept *in vivo* experiment was performed in Panc 10.05 PTEN KO xenografts, a useful but suboptimal tool, that doesn’t capture the complexity of spontaneous resistance elicited by the selective pressure of treatment *in vivo*. Therefore, further validation in more clinically relevant mouse models (genetically engineered or patient-derived xenografts) will be needed to support the clinical translation of our findings. Nevertheless, the results of our proof-of -concept *in vivo* experiment are in line with our previous findings that identified MAP2K4 as a central mediator of feedback activation of JNK–JUN in response to MAPK inhibition [55], as well as with our demonstration that MAP2K4 inhibition, when paired with MEK, KRAS-G12C or pan-RAS blockade, yields durable tumor regressions in KRAS-mutant lung and colon xenografts [56]. In this context, the clinical grade MAP2K4 inhibitor HRX-0215 appears as an interesting therapeutic companion to MAPK inhibitors, as previous clinical trials for liver regeneration have shown favorable tolerability [57].

Direct targeting of RAS — previously considered undruggable — has recently become a realistic and promising strategy [5, 58, 59]. The RAS(ON) multi-selective inhibitor RMC-6236, for instance, inhibits both wild-type and mutant forms by blocking their active GTP-bound state, yielding potent and broad antiproliferative effects in KRAS-mutant preclinical PDAC models [59]. Recently, combination strategies employing SHP2 and RAS-G12C(ON) inhibition have been explored in the context of lung cancer, where it was shown to sensitize tumors to immune checkpoint blockade [60]. We explored a similar strategy, but with the use of RAS(ON) multi-selective inhibitors, in the context of KRAS-mutant pancreatic cancer, and we showed that PDAC cell lines respond synergistically to the combination of SHP2 + RAS(ON) multi-selective, comparably to the effect observed with SHP2 + ERK inhibitors. We then asked whether the same mechanisms of resistance identified for the combination of SHP2 and ERK inhibitors would confer resistance also to SHP2 plus RAS(ON) multi-selective inhibitors. By generating and analyzing spontaneous MiaPaCa-2 resistors, we observed a decrease in PTEN protein levels and an upregulation of JUN, similar to what we observed in the case of resistance to SHP2 + ERK inhibitors. Also, we showed that JUN overexpression is sufficient to confer resistance to SHP2 plus RAS(ON) multi-selective inhibitors. Interestigly, JUN amplification was also reported in one PDAC mouse that developed resistance to the monotherapy with the RAS(ON) multi-selective inhibitor RMC-7977 (preclinical equivalent of RMC-6236) [59].

## Conclusions

Overall, our study reveals a previously underrated convergence of the mTOR and JUN axis in mediating resistance to combined SHP2 and ERK or RAS(ON) inhibitor therapy in PDAC. It also indicates that JUN expression or phosphorilation could be used as predictive biomarkers for resistance, guiding patients selection in relevant clinical trials, and indicates indirect JUN suppression through MAP2K4 inhibitors as a possible pharmacological strategy to overcome such resistance.

## Supplementary Information

Additional file 1. SupplementaryFigures.pdf contains Supplementary Figures 1-6.

Additional file 2. SupplementaryFigureLegends.docx contains the legends to the Supplementary Figures included in the Additional File 1.

Additional file 3. Panc10.05 PTEN KO tumor volumes.xlsx contains the tumor volumes relative to the xenograft experiment reported in Figure 4A.

## Data Availability

The dataset used to generate Fig. 3 C, and supporting the conclusions of this article, is available in the GEO repository, under accession number GSE303578, [https://www.ncbi.nlm.nih.gov/geo/query/acc.cgi?acc = GSE303578] (https://www.ncbi.nlm.nih.gov/geo/query/acc.cgi?acc = GSE303578). The tumor volumes of the *in vivo* experiment reported in Fig. 4 A are available in Additional File 3. Further materials supporting the results presented in this paper are available upon reasonable request to the corresponding authors.

## Abbreviations

AKT: Protein kinase B
AP1: Activator protein 1
ATCC: American type culture collection
BATF: Basic leucine zipper ATF-like transcription factor
BCA: Bicinchoninic acid
Cas9: CRISPR-associated protein 9
CCLE: Cancer cell line encyclopedia
COP1/RFWD2: Constitutive photomorphogenesis protein 1
COSMIC: Catalogue of somatic mutations in cancer
CRISPR: Clustered regularly interspaced short palindromic repeats
CUL4: Cullin 4
CYB5R4: Cytochrome b5 reductase 4
DDB1: DNA damage binding protein 1
DET1: De-etiolated homolog 1
EGFR: Epidermal growth factor receptor
ERK: Extracellular signal-regulated kinase
FACS: Fluorescence-associated cell sorting
FBS: Fetal bovine serum
FDA: Food and drug administration
FDR: False discovery rate
FOS: Fos proto oncogene AP-1 transcription factor subunit
GAPDH: Glyceraldehyde-3-phosphate dehydrogenase
GFP: Green fluorescence protein
JNK: C-Jun N-terminal kinase
JUN: C-Jun
KO: Knock out
KRAS: Kirsten rat sarcoma viral oncogene homolog
MAP2K4: Mitogen-activated protein kinase kinase 4
MAPK: Mitogen-activated protein kinase
MEK: Mitogen-activated protein kinase kinase
MOI: Multiplicity of infection
mTOR: Mammalian target of rapamycin
mTORC1: Mammalian target of rapamycin complex 1
NSCLC: Non-small cell lung cancer
NSG: NOD scid gamma
PARP: Poly (ADP-ribose) polymerase
PCR: Polymerase chain reaction
PDAC: Pancreatic ductal adenocarcinoma
PDCD10: Programmed cell death 10
PI3K: Phosphoinositide 3-kinase
PPP2R4: Protein phosphatase 2A regulatory subunit 4
PPP6C: Protein phosphatase 6 catalytic subunit
PTEN: Phosphatase and TENsin homolog deleted on chromosome 10
RAF: Rapidly accelerated fibrosarcoma
RIPA: Radioimmunoprecipitation assay
RRA: Robustrank algorithm
RSK1: Ribosomal S6 kinase 1
RTK: Receptor tyrosine kinase
S6RP: S6 rybosomal protein
sgRNA: Single guide RNA
SHP2: Src homology region 2 (SH2)-containing protein tyrosine phosphatase-2
SOS1: Son of sevenless homolog 1
STR: Short tandem repeats
TIPRL: TOR signaling pathway regulator-like
