# Supplementary material for: CRISPR knockout screens reveal JUN as the master mediator of resistance to MAPK inhibition in KRAS-mutant pancreatic cancer
Source: J Exp Clin Cancer Res. 2026 Jan 22;45:60. doi: 10.1186/s13046-025-03616-z (PMC12947432; doi:10.1186/s13046-025-03616-z)

Supplementary Figure 1

A

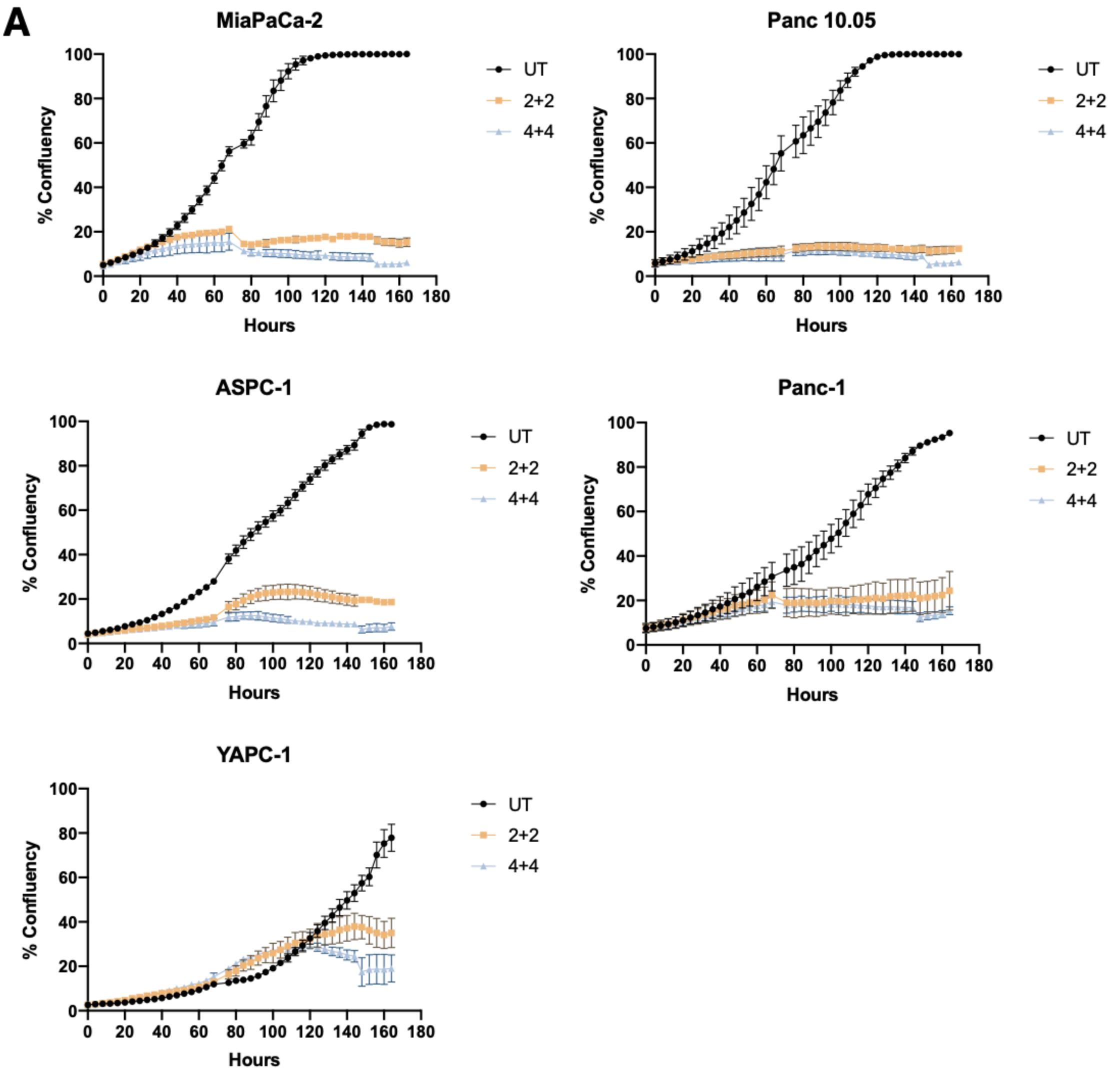

B

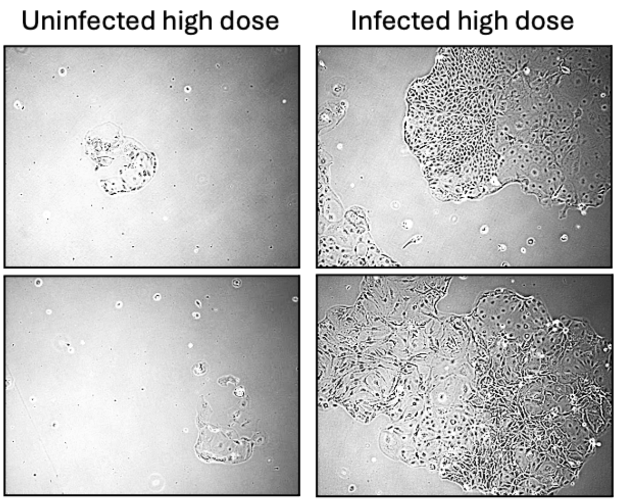

C

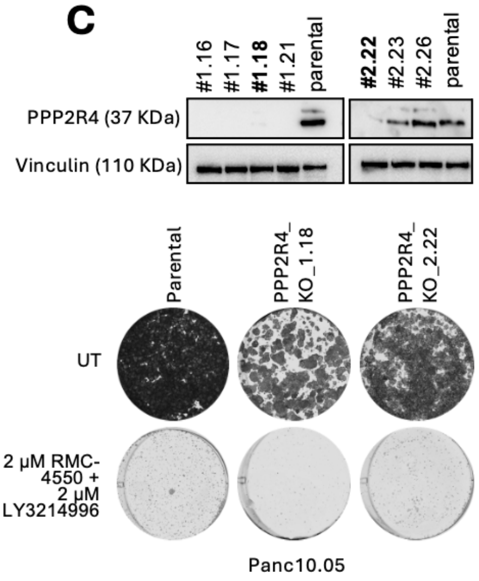

Supplementary Figure 2

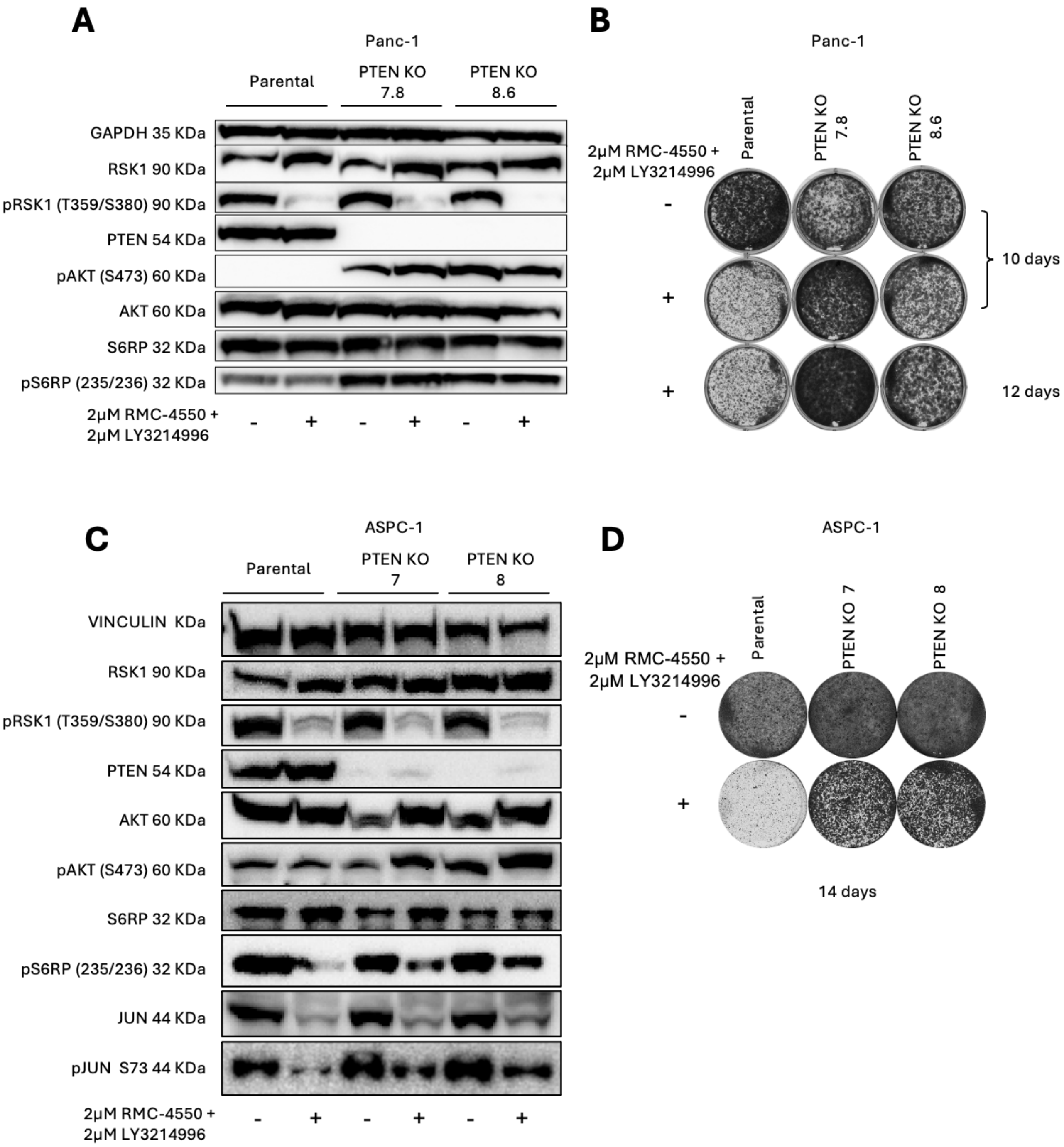

Supplementary Figure 3

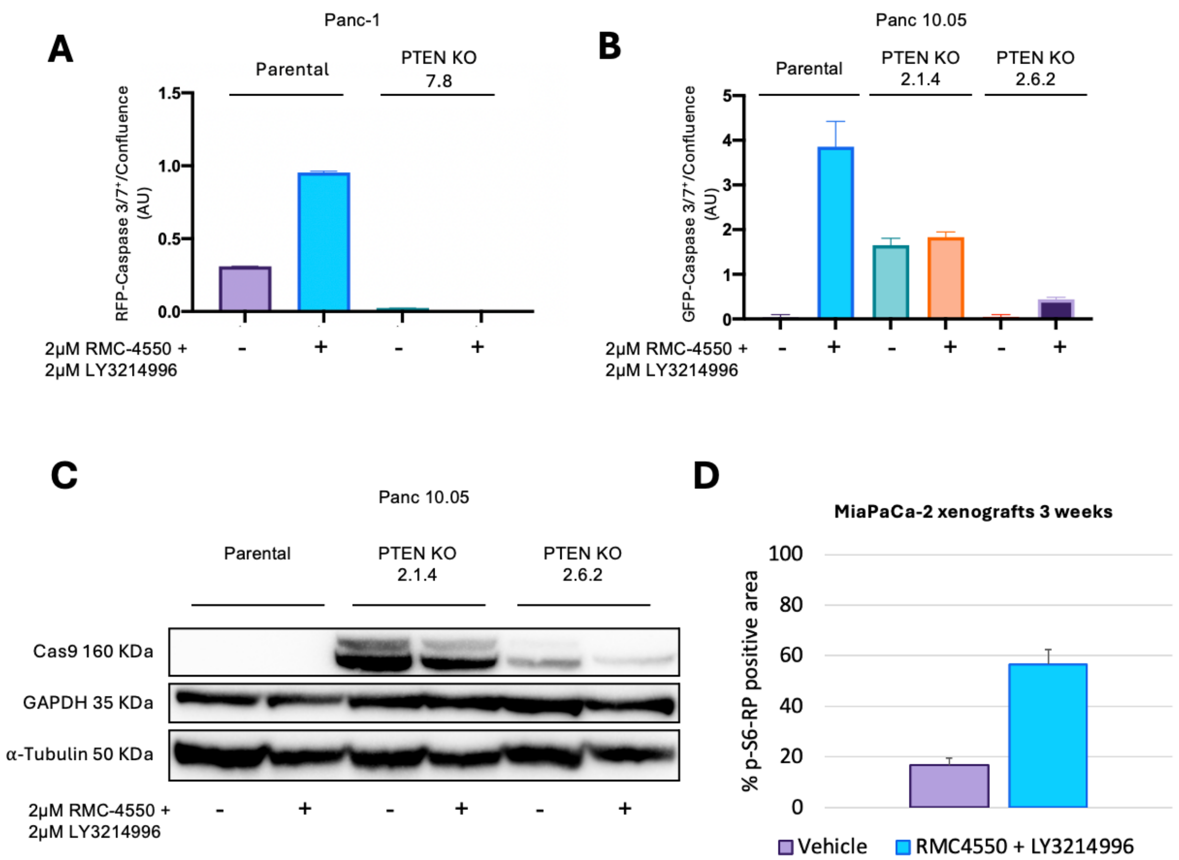

Supplementary Figure 4

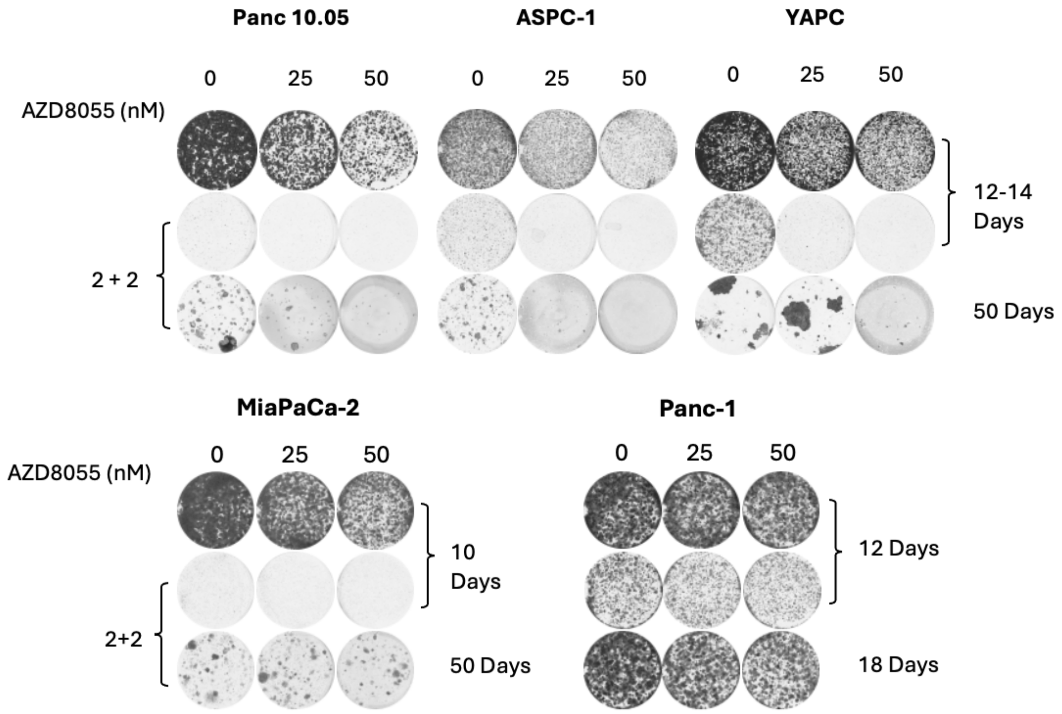

Supplementary Figure 5

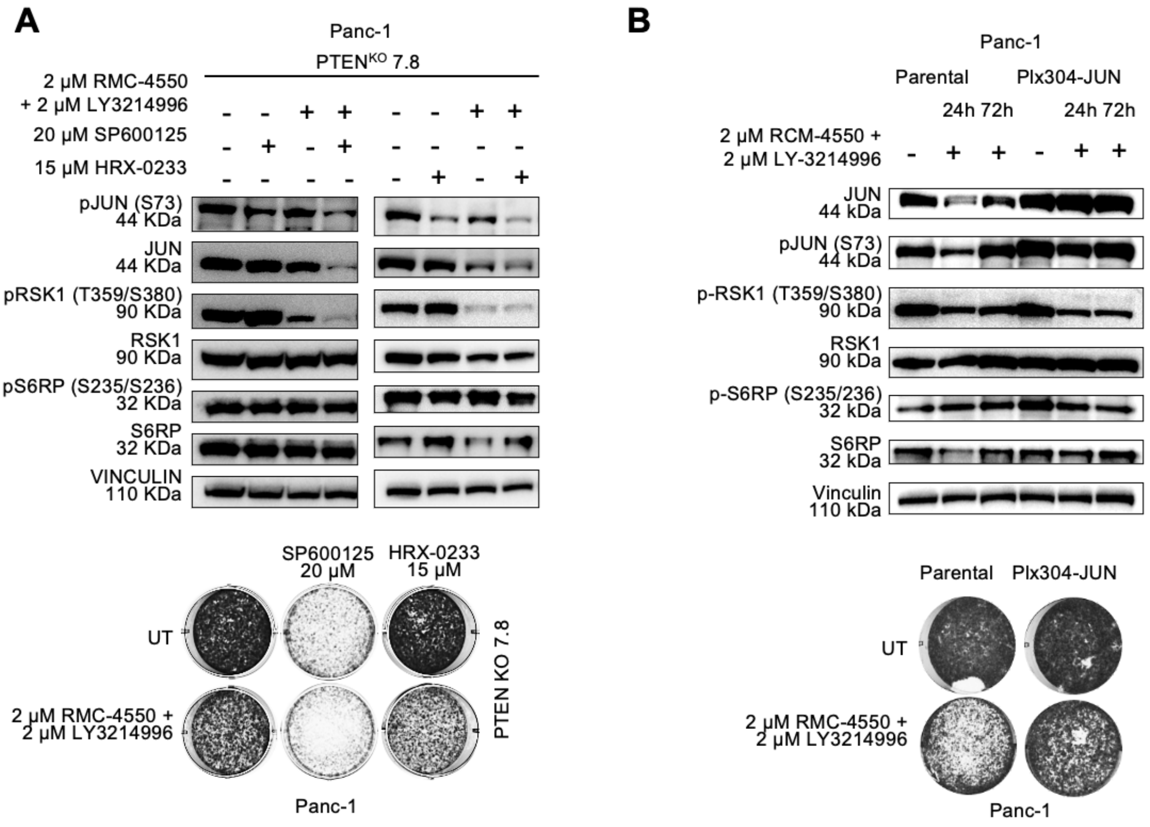

Supplementary Figure 6

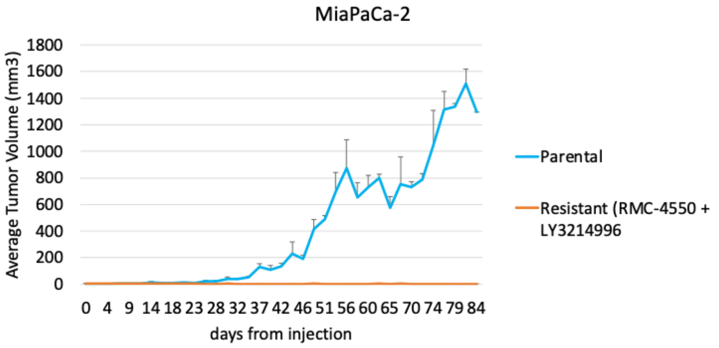

Supplementary Figure 7

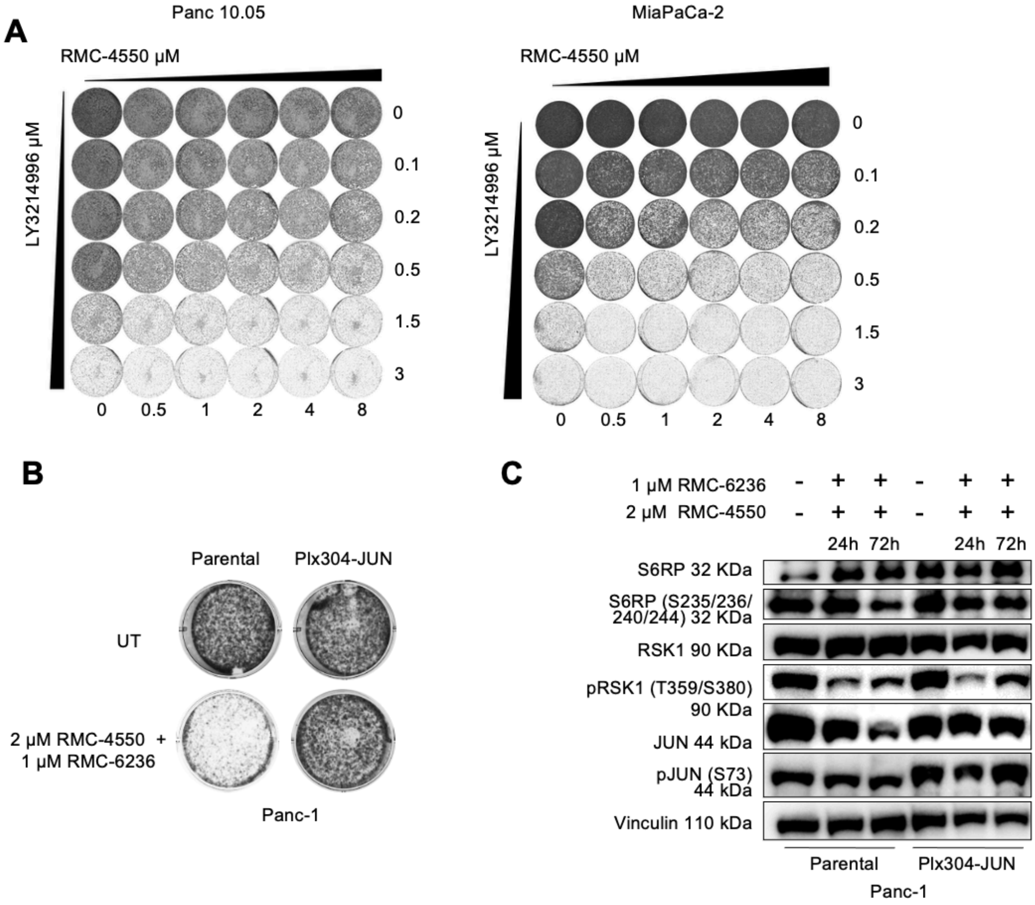

Supplement: Supplementary file 1 — Additional file 1. SupplementaryFigures.pdf contains Supplementary Figures 1-6. [file 13046_2025_3616_MOESM1_ESM.pdf]
