# Supplementary material for: CRISPR knockout screens reveal JUN as the master mediator of resistance to MAPK inhibition in KRAS-mutant pancreatic cancer
Source: J Exp Clin Cancer Res. 2026 Jan 22;45:60. doi: 10.1186/s13046-025-03616-z (PMC12947432; doi:10.1186/s13046-025-03616-z)
Supplement: Supplementary file 2 — Additional file 2. SupplementaryFigureLegends.docx contains the legends to the Supplementary Figures included in the Additional File 1. [file 13046_2025_3616_MOESM2_ESM.docx]

**Supplementary Figure Legends**

**Supplementary Figure 1. A:** Incucyte® growth curves, where percentage of confluency was measured over time for 160 hours. Conditions: untreated (UT, black line), 2 μM LY3214996 plus 2 μM RMC-4550 (“2+2”, orange line) and 4 μM LY3214996 plus 4 μM RMC-4550 (“4+4”, blue line). Cell lines: MiaPaCa-2, Panc 10.05, ASPC-1, Panc-1 and YAPC-1. Averages of n=3 replicates are shown, error bars indicate standard deviations. **B**: Representative images of resiatant colonies arising from cells that were infected or not with the CRISPR Brunello genome wide library after 23 days of culture. **C**: upper panel, western blot analysis of Panc 10.05 cells parental or PPP2R4 KO single-cell clones, to evaluate the level of knockout. Lysates were probed with antibodies against PPP2R4 and vinculin as a loading control; lower panel, long term proliferation assay of Panc10.05 parental or PPP2R4 KO clones 1.18 and 2.22 either untreated or treated with the combination of RMC-4550 plus LY3214996. After 14 days, cells were fixed and stained with crystal violet.

**Supplementary Figure 2. A:** western blot analysis of Panc-1 parental and PTEN KO clones 7.8 and 8.6, either untreated or treated with 2 μM LY3214996 and 2 μM RMC-4550 for 48 hours. RSK1 and pRSK1 serve as read out of the MAPK pathway activity. PTEN, AKT, pAKT, S6RP and pS6RP serve as read out of the PI3K-AKT-mTOR axis activity. GAPDH serves as loading control. Representative image of at least 3 independent experiments giving similar results. **B**: long term proliferation assay of Panc-1 parental or PTEN KO clones 7.8 and 8.6 either untreated or treated with the combination of 2 μM LY3214996 plus 2 μM RMC-4550. After 10 or 12 days, cells were fixed and stained with crystal violet. Representative images of at least 3 independent experiments giving similar results. **C**: western blot analysis of ASPC-1 parental and PTEN KO polyclonal populations (sgRNA 7 or sgRNA 8), either untreated or treated with 2 μM LY3214996 and 2 μM RMC-4550 for 24 hours. RSK1 and pRSK1 serve as read out of the MAPK pathway activity. PTEN, AKT, pAKT, S6RP and pS6RP serve as read out of the PI3K-AKT-mTOR axis activity. JUN and pJUN represent the most downtream nodes of the pathway. GAPDH serves as loading control. Representative image of at least 3 independent experiments giving similar results. **B**: long term proliferation assay of ASPC-1 parental or PTEN KO polyclonal populations (sgRNA 7 or sgRNA 8) either untreated or treated with the combination of 2 μM LY3214996 plus 2 μM RMC-4550. After 6 days, cells were fixed and stained with crystal violet. Representative images of at least 3 independent experiments giving similar results.

**Supplementary Figure 3. A**: RFP-Caspase 3/7 apoptosis assay, measuring at 72h the levels of RFP in Panc-1 parental and PTEN KO 7.8. cells. Cells were either untreated or treated with 2 μM LY3214996 plus 2 μM RMC-4550. The bars represent the average of n=3 replicates. Error bars represent standard deviation. **B**: GFP-Caspase 3/7 apoptosis assay, measuring at 72h the levels of GFP in Panc 10.05 parental and PTEN KO 2.1.4 and 2.6.2 clones. Cells were either untreated or treated with 2 μM LY3214996 plus 2 μM RMC-4550. The bars represent the average of n=3 replicates. Error bars represent standard deviation. **C**: western blot analysis showing the expression levels of Cas9 in Panc 10.05 parental cells and PTEN KO 2.1.4 and 2.6.2 clones. GAPDH and alpha-tubulin serve as loading controls. **D**: quantification of IHC for pS6RP. Bars represent the average percentage of positive area over 3 sections from 3 different mice. Error bars represent standard deviation.

**Supplementary Figure 4.**Long term proliferation assay for Panc 10.05, ASPC-1, YAPC-1 (upper panel), MiaPaCa-2 and Panc-1 (lower panel). Cells were treated with increasing doses of AZD8055 (0, 25, 50 nM) as monotherapy (upper row) or in combination with 2 μM LY3214996 and 2 μM RMC-4550 (“2+2”) (middle and lower rows). Cells were treated for a short timepoint (10 to 14 days as indicated) until the untreated dish (0) reached confluency, or for for a long timepoint (18 or 50 days as indicated).

**Supplementary Figure 5. A:** upper panel, western blot analysis of Panc-1 PTEN KO clone 7.8 either untreated, treated with the JNK inhibitor SP600125 (15 μM) , the MAP2K4 inhibitor HRX-0233 (15 μM), the combination of 2 μM LY3214996 plus 2 μM RMC-4550, or one of the two triple combinations (LY3214996 plus RMC-4550 plus SP600125 or LY3214996 plus RMC-4550 plus HRX-0233) for 72 hours. Lysates were probed with antibodies against JUN, s6RP and RSK1 (all total and phosphorylated). Vinculin served as loading control; lower panel, long term proliferation assay of Panc-1 PTEN KO clone 7.8 either untreated, treated with the JNK inhibitor SP600125 (15 μM) , the MAP2K4 inhibitor HRX-0233 (15 μM), the combination of 2 μM LY3214996 plus 2 μM RMC-4550, or one of the two triple combinations (LY3214996 plus RMC-4550 plus SP600125 or LY3214996 plus RMC-4550 plus HRX-0233) for 14 days, after which the cells were fixed and stained with crystal violet. Images are representative of 3 independent experiments giving similar results. **B**: upper panel, western blot analysis of Panc-1 parental and JUN overexpressing (Plx304-JUN) cells either untreated or treated with the combination of 2 μM LY3214996 plus 2 μM RMC-4550 for 24 or 72 hours as indicated. Lysates were probes with primary antibodies against JUN, RSK1 and S6RP (all total and phosphorylated). Vinculin served as a loading control. Images are representative of 3 independent experiments giving similar results; lower panel, long term proliferation assay of Panc-1 parental and JUN overexpressing (Plx304-JUN) cells, either untreated or treated with the combination of 2 μM LY3214996 plus 2 μM RMC-4550 for 14 days. After this time, cells were fixed and stained with crystal violet. Images are representative of 3 independent experiments giving similar results.

**Supplementary Figure 6.** Engrafting experiment for the MiaPaCa-2 parental and resistant (to SHP2 plus ERK inhibitors). 5 × 10^6^ cells were subcutaneously injected into the right flank of NSG mice, and tumor volume was followed over time. N= 4 mice per group. Mean tumor volumes ± s.e.m. are shown.

**Supplementary Figure 7. A:** Long term proliferation assay of Panc 10.05 and MiaPaCa-2 cells performed in a matrix of increasing concentrations of SHP2 inhibitor (RMC-4550) and ERK inhibitor (LY3214996) and their combination for 6 days. At the end, cells were fixed and stained with crystal violet. Images are representative of 3 independent experiments giving similar results **B:** long term proliferation assay of Panc-1 parental and JUN overexpressing (Plx304-JUN) cells, either untreated or treated with the combination of 2 μM RMC-4550 plus 1 μM RMC-6236 for 14 days. After this time, cells were fixed and stained with crystal violet. Images are representative of 3 independent experiments giving similar results. **C:** western blot analysis of Panc-1 parental and JUN overexpressing (Plx304-JUN) cells either untreated or treated with the combination of 2 μM RMC-4550 plus 1 μM RMC-6236 for 24 or 72 hours as indicated. Lysates were probes with primary antibodies against JUN, RSK1 and S6RP (all total and phosphorylated). Vinculin served as a loading control. Images are representative of 3 independent experiments giving similar results.
